# A Comparative Analysis of Women’s Experiences with Cone Beam Breast CT and Digital Mammography in Australia

**DOI:** 10.3390/jcm15208085

**Published:** 2026-10-18

**Authors:** Kelly Bentley-Spuur, Clare L. Singh, Minh T. Chau

**Affiliations:** 1 School of Dentistry and Medical Sciences, Faculty of Science and Health, Charles Sturt University, P.O. Box 588, Wagga Wagga, NSW 2650, Australia; csingh@csu.edu.au (C.L.S.); schau@csu.edu.au (M.T.C.); 2 Teaching Innovation in Radiography Group, School of Dentistry and Medical Sciences, Faculty of Science and Health, Charles Sturt University, P.O. Box 588, Wagga Wagga, NSW 2650, Australia; 3 College of Health Sciences, Vin University, Vinhomes Ocean Park, Gia Lam Commune, Ha Noi 12400, Vietnam; 4 School of Medical and Health Sciences, Tung Wah College, Hong Kong, China

**Keywords:** cone beam breast CT, CBBCT, mammography, breast cancer, diagnostic mammography, breast screening, breast imaging, compression

## Abstract

**Background/Objectives:** Cone-beam breast computed tomography (CBBCT) is a promising alternative to digital mammography (mammography), producing true 3D images without breast manipulation or compression. This technological difference is expected to improve patient comfort and reduce anxiety-related imaging barriers. This paper explores women’s experiences comparing CBBCT and mammography in Australia. **Methods:** A survey at Brisbane Radiology, Queensland, Australia evaluated women’s experiences of mammography and CBBCT, including perceptions of comfort, pain, positioning, anxiety, satisfaction, perceived diagnostic confidence, and future breast imaging preferences. Quantitative data were analysed using descriptive statistics; open-ended responses underwent reflexive thematic analysis. **Results:** Of 42 participants, the majority 30/42 (71.4%) found mammography difficult, uncomfortable 30/42 (71.4%), or dissatisfying 21/42 (50%), with 20/42 (47.6%) having previously stopped mammography due to pain (16/20) 80% or fear of compression (11/20) 55%. Conversely, women chose CBBCT primarily for its lack of compression 36/42 (85.7%) and perceived diagnostic accuracy 28/42 (66.7%). Most rated CBBCT as comfortable 33/42 (78.6%), reported high overall satisfaction 39/42 (92.9%), and preferred it for future imaging 36/42 (85.7%). Qualitative analysis identified five themes: (1) mammography-associated pain and avoidance, (2) CBBCT-associated comfort and dignity, (3) improved confidence in diagnostic accuracy, (4) the value of supportive staff, (5) accessibility concerns. **Conclusions:** The findings reinforce that mammography causes significant discomfort, pain, and anxiety. In contrast, CBBCT provides a positive, well-tolerated, and compression-free alternative to traditional breast imaging pathways. The small single-site sample reflects data from the only CBBCT unit in Australia, which limits the generalizability of the results. A larger sample size is needed to validate the results of this study.

## 1. Introduction

Breast cancer remains a leading cause of cancer-related morbidity and mortality among women globally [1]. In Australia, breast cancer is the most diagnosed female cancer, with an estimated incidence of 140 cases per 100,000 in 2025 [2]. Mammography is currently the gold standard imaging modality for breast cancer diagnosis in the diagnostic setting and stands as the cornerstone of early breast cancer detection in global screening efforts. Where organised mammography screening programs have been implemented, these programs have demonstrably reduced mortality through pre-clinical detection and treatment [3,4].

However, despite its proven efficacy, full participation in the Australian BreastScreen programs remains unrealized, with only 52% of eligible women engaging with the program in 2023–2024 [5]. Participation remains one of the key determinants of screening program effectiveness [6]. Previous studies have demonstrated that up to one-third of eligible women do not attend their first screening examination, which is associated with lower ongoing adherence and increased risk of late-stage diagnosis and mortality [7]. Barriers to breast screening participation are well recognised and are often directly related to the physical and psychological experience of mammography itself. Commonly reported barriers include breast manipulation, positioning discomfort, compression-related pain, anxiety, embarrassment, and previous negative imaging experiences [8].

Patients attending for diagnostic mammography have been referred for imaging due to breast symptoms or are being routinely imaged due to significant family history. Their drivers and motivators to undergo imaging are very different from those attending for routine screening; however, breast manipulation, positioning discomfort, compression-related pain, anxiety, embarrassment, and previous negative imaging experiences prevail.

Cone-beam breast computed tomography (CBBCT) has emerged as a promising alternative to conventional screening and diagnostic mammography and has been developed to address several of these known issues [9]. Unlike mammography, CBBCT produces true three-dimensional breast imaging without requiring breast manipulation or compression. This technological difference is expected to improve patient comfort and reduce anxiety-related barriers associated with breast imaging participation.

Clinical studies and meta-analyses have demonstrated encouraging diagnostic performance of CBBCT for breast cancer detection, with pooled sensitivity and specificity reported at 0.95 (95%CI: 0.91–0.97) and 0.72 (95%CI: 0.62–0.80), respectively, and an area under the curve of 0.92 (95%CI: 0.90–0.94) [10]. Additionally, CBBCT facilitates improved visualisation of pathology, including breast cancer, in dense breast tissue, which remains a recognised and significant limitation of mammography, suggesting potential technical advantages over traditional mammographic imaging [11]. Despite these advantages, CBBCT has not yet been widely adopted into routine clinical practice. Existing literature has largely focused on technical performance and diagnostic accuracy, with relatively limited attention given to women’s experiences and perceptions of the technology. Whilst one study reported increased patient comfort with CBBCT compared to digital mammography (*p* < 0.05) [12], there remains limited evidence exploring the acceptability of CBBCT from a person-centred perspective or examining whether the technology may mitigate barriers associated with mammography participation and imaging adherence.

Improved understanding of women’s experiences of CBBCT may inform future breast imaging pathways, screening strategies, and implementation considerations, particularly among women who have previously delayed or discontinued mammography due to pain, anxiety, or previous negative experiences.

The primary aim of this study was to explore Australian women’s experiences of CBBCT imaging in comparison with screening and diagnostic mammography. Secondary aims were to examine differences in perceived comfort and satisfaction between CBBCT and mammography, explore reasons for anxiety associated with both examinations, identify women’s preferred breast imaging modality, and investigate factors influencing engagement with CBBCT among women who had previously discontinued mammography. It was hypothesised that women would report greater comfort, lower anxiety, and higher satisfaction with CBBCT compared with mammography, and that women who had previously ceased mammographic imaging would report CBBCT as a more acceptable breast imaging modality.

## 2. Materials and Methods

### 2.1. Study Design

This study employed a cross-sectional mixed-methods survey design to explore women’s experiences of CBBCT imaging in comparison with digital mammography (mammography). The study incorporated both quantitative and qualitative components to capture women’s perceptions of comfort, pain, positioning, anxiety, satisfaction, perceived diagnostic confidence, and future breast imaging preferences following CBBCT imaging.

A mixed-methods approach was selected to enable quantitative evaluation of participant experiences while also facilitating deeper exploration of women’s personal narratives and reflections regarding mammography and CBBCT imaging. The inclusion of open-ended questions allowed participants to elaborate on previous breast imaging experiences, reasons for delaying or discontinuing mammography, perceived advantages and disadvantages of CBBCT, and recommendations for future implementation of the technology.

The study was conducted according to the guidelines of the Declaration of Helsinki and approved by the Institutional Review Board/Ethics Committee of Charles Sturt University Human Research Ethics Committee, Protocol number: H25368, on 6 November 2025.

### 2.2. Setting and Participants

The study was conducted in collaboration with Brisbane Radiology, Queensland, Australia, where CBBCT imaging had been introduced into clinical practice in July 2025. Participants were women who had undergone bilateral CBBCT imaging at this same radiology practice between January 2026 and March 2026.

Eligible participants were women aged 18 years or older who had previous experience with mammography (screening or diagnostic) and subsequently underwent CBBCT imaging during the study period.

Patients were excluded if they had never previously undergone mammography, were male, or submitted incomplete survey responses. A convenience sampling approach was used, with the final sample reflecting a subset of 469 women who underwent CBBCT imaging between July 2025 and 1 March 2026 and elected to participate in the survey.

### 2.3. Survey Instrument and Data Collection

Data were collected using a purpose-designed online survey created and administered via Qualtrics (Provo, UT, USA), which was reviewed by industry experts prior to being administered. The survey was available between 28 January and 2 March 2026 and consisted of both closed-ended and open-ended questions and was designed to evaluate participants’ experiences of mammography and CBBCT imaging. The survey initially screened participants using eligibility questions and incorporated skip logic to direct women to appropriate question pathways. Participants were categorised into three groups: women who had never undergone mammography, who were excluded from participation; women who had previous mammography experience but had consciously ceased mammographic imaging prior to attending CBBCT; and women who had continued mammographic imaging prior to CBBCT. Participants who reported discontinuing mammography were directed to additional questions exploring their reasons for ceasing mammography, factors influencing their decision to return to imaging, and experiences of CBBCT.

The questionnaire consisted of seven sections examining demographic and background characteristics, reasons for discontinuing mammography, previous mammography experiences, experiences of CBBCT imaging, comparative perceptions between mammography and CBBCT, perceptions among women who had ceased mammography, and additional open-ended feedback.

Quantitative items explored demographic characteristics, mammography history, breast cancer history, previous breast surgery, breast implants, family history of breast cancer, comfort, pain, positioning, anxiety, satisfaction, confidence in imaging results, and future imaging preferences. Open-ended questions enabled participants to provide narrative reflections regarding their imaging experiences, concerns, perceptions of diagnostic confidence, accessibility issues, and recommendations for future implementation of CBBCT technology.

### 2.4. Quantitative Data Analysis

Quantitative survey data were exported into Microsoft Excel (Microsoft Corporation, Redmond, WA, USA) before being analysed using IBM SPSS Statistics for Windows Version 26 (IBM Corp., Armonk, NY, USA).

Descriptive statistics were used to summarise participant demographic characteristics and survey responses. Categorical variables were reported as frequencies and percentages. Exploratory subgroup comparisons examined differences between participants with previous diagnostic mammography experience (*n* = 27) and those with previous routine BreastScreen mammography experience (*n* = 15).

Two-sided Fisher’s exact tests compared the proportions reporting each outcome, given the small subgroup sizes and sparse cell counts. Each reported response category was compared with all remaining responses. Unadjusted *p*-values were reported, and the Holm procedure was applied across the 20 comparisons to account for multiple testing. Statistical significance was assessed using Holm-adjusted *p*-values below 0.05.

### 2.5. Qualitative Data Analysis

Responses to open-ended survey questions were analysed using reflexive thematic analysis informed by Braun and Clarke’s six-phase approach to develop themes through sustained engagement with the data, researcher interpretation, and iterative reflection on participants’ experiences and meanings [13]. Qualitative responses were initially reviewed repeatedly by members of the research team to achieve familiarity with the dataset and identify recurrent patterns across participant narratives.

Initial codes were generated inductively from participant responses before being iteratively grouped into broader categories and themes. Codes and emerging themes were reviewed collaboratively among the research team to enhance interpretive rigour and consistency.

Themes were refined through repeated engagement with the dataset and developed to reflect recurrent participant experiences and perceptions regarding mammography and CBBCT imaging. Representative participant quotations were selected to illustrate each theme and preserve participants’ perspectives within the analysis. The complete raw dataset is available in Table S1.

## 3. Results

### 3.1. Participant Demographics and Clinical Characteristics

A total of 42 participants from both the diagnostic and routine screening settings were included in the final analysis following the exclusion of four women who had never previously undergone mammography and three incomplete survey responses. Participant demographic and clinical characteristics are presented in Table 1. 

### 3.2. Mammography Experiences

Mammography was frequently described as uncomfortable and painful across both cohorts. Participant-reported mammography experiences are summarised in Table 2. 

**Table 2:** Previous mammography experiences among participants.

Mammography Experience Variable	Total n (%)	Diagnostic Setting n (%)	Routine Screening n (%)	Fisher’s *p*
Total participants	42 (100)	27 (64.3)	15 (35.7)	
Difficult or very difficult positioning	30 (71.4)	19 (70.4)	11 (73.3)	1.000
Uncomfortable or very uncomfortable mammography	30 (71.4)	18 (66.7)	12 (80.0)	0.485
Moderate-to-extreme pain/discomfort	31 (73.8)	23 (85.2)	8 (53.3)	0.034
Severe or extreme pain/discomfort	14 (33.3)	11 (40.7)	3 (20.0)	0.306
Unsatisfied or very unsatisfied with mammography	21 (50.0)	15 (55.6)	6 (40.0)	0.520
Anxiety before or during mammography	29 (69.0)	20 (74.1)	9 (60.0)	0.488
Concern regarding imaging accuracy	22 (52.4)	16 (59.3)	6 (40.0)	0.336
Previous bad experience with compression	17 (40.5)	12 (44.4)	5 (33.3)	0.531
Fear of compression	11 (26.2)	8 (29.6)	3 (20.0)	0.717
Concern regarding cancer diagnosis/recurrence	15 (35.7)	11 (40.7)	4 (26.7)	0.506

Footnotes: *p*-values are from two-sided Fisher’s exact tests comparing diagnostic and routine screening subgroups. Holm correction was applied across all 20 comparisons in this table and Table 3. No comparison remained statistically significant after adjustment.

**Table 3:** Cone beam breast CT imaging experiences and preferences.

Cone Beam Breast CT Variable	Total n (%)	Previous Imaging Diagnostic Setting n (%)	Previous Imaging Routine Screening n (%)	Fisher’s *p*
Total participants	42 (100)	27 (64.3)	15 (35.7)	
Comfortable or very comfortable examination	33 (78.6)	21 (77.8)	12 (80.0)	1.000
None or mild pain/discomfort during CBBCT	36 (85.7)	22 (81.5)	14 (93.3)	0.395
Satisfied or very satisfied with CBBCT	39 (92.9)	24 (88.9)	15 (100)	0.541
No concerns before or during CBBCT	29 (69.0)	18 (66.7)	11 (73.3)	0.739
Would undergo CBBCT again in future	41 (97.6)	26 (96.3)	15 (100)	1.000
Reported CBBCT was “much better” than mammography	37 (88.1)	24 (88.9)	13 (86.7)	1.000
Identified CBBCT as more comfortable	38 (90.5)	25 (92.6)	13 (86.7)	0.608
Felt more relaxed during CBBCT	36 (85.7)	23 (85.2)	13 (86.7)	1.000
Preferred CBBCT for future breast imaging	36 (85.7)	24 (88.9)	12 (80.0)	0.649
Believed CBBCT should be more widely available	40 (95.2)	27 (100)	13 (86.7)	0.122

Footnotes: *p*-values are from two-sided Fisher’s exact tests comparing diagnostic and routine screening subgroups. Holm correction was applied across all 20 comparisons in Table 2 and this table. No comparison remained statistically significant after adjustment.

### 3.3. Reasons for Discontinuing Mammography

Twenty participants (47.6%) reported consciously discontinuing or delaying mammography prior to undergoing CBBCT imaging (Table 1). Overall, 16/20 (80.0%) stopped due to pain and 11/20 (55.0%) due to fear of compression.

Among those who had undergone previous diagnostic mammography, the most frequently reported reasons for discontinuation included pain or discomfort during mammography (11/14, 78.6%), fear of compression (8/14, 57.1%), concerns regarding imaging accuracy (7/14, 50.0%), and anxiety or emotional distress (5/14, 35.7%). Several participants also described concerns regarding dense breast tissue, prior traumatic imaging experiences, and previous negative interactions with imaging staff.

Of those who had undergone previous routine screening, pain or discomfort during mammography was reported by 5/6 (83.3%) participants who had ceased imaging, while fear of compression was reported by 3/6 (50.0%). Concerns regarding diagnostic accuracy or lack of confidence in mammography were reported by 4/6 (66.7%) women.

Several women described previous mammography experiences as traumatic, particularly among participants with dense breasts, previous surgery, breast implants, or prior recalls.

### 3.4. Reasons for Attending Cone Beam Breast CT Imaging

The most common reasons for attending CBBCT imaging were the perception that the examination was more comfortable than mammography, the absence of breast compression, and increased confidence in diagnostic accuracy.

Of the participants who had previously undergone diagnostic mammography, 22/27 (81.5%) participants selected “it does not involve breast compression” as a reason for attending CBBCT imaging, while 18/27 (66.7%) reported believing the technology was more accurate. Sixteen participants (59.3%) reported feeling safer or more confident in the imaging results.

Of those who had undergone previous routine screening, 12/15 (80.0%) participants selected “it does not involve breast compression,” and 10/15 (66.7%) believed the technology was more accurate than mammography. Nine participants (60.0%) reported feeling safer or more confident in the imaging results.

Healthcare providers were the most common source of information about CBBCT across both cohorts, reported by 20/42 participants (47.6%), followed by family or friends (8/42, 19.0%), television or news media (7/42, 16.7%), online sources (4/42, 9.5%), and social media (3/42, 7.1%).

### 3.5. Cone Beam Breast CT Imaging Experiences

Overall experiences with CBBCT imaging were highly positive. Participant-reported CBBCT experiences and imaging preferences are summarised in Table 3.

Of those who had undergone previous diagnostic mammography, 21/27 (77.8%) participants rated CBBCT imaging as comfortable or very comfortable. Twenty-two participants (81.5%) reported no pain or only mild pain during the examination. Twenty-four participants (88.9%) reported being satisfied or very satisfied with the overall CBBCT experience.

Similarly, of those who had previously undergone routine screening, 12/15 (80.0%) of participants described the examination as comfortable or very comfortable. Fourteen participants (93.3%) reported no pain during imaging. All 15 participants (100%) reported being satisfied or very satisfied with the overall CBBCT experience. Most participants described positioning on the CBBCT table as easy or very easy, although some women identified difficulties related to ladder access, shoulder discomfort, and positioning for women with mobility limitations.

### 3.6. Comparison Between Mammography and Cone Beam Breast CT

Participants overwhelmingly preferred CBBCT over mammography. Of those who had undergone previous diagnostic mammography, 24/27 (88.9%) participants described the overall experience as “much better” than mammography. Twenty-five participants (92.6%) identified CBBCT as the more comfortable imaging modality, while 23/27 (85.2%) reported feeling more relaxed during CBBCT imaging. Twenty-four participants (88.9%) preferred CBBCT for future breast imaging if both modalities were available.

Of those who had previously undergone routine screening, 13/15 (86.7%) participants described CBBCT as “much better” than mammography. Thirteen participants (86.7%) reported that CBBCT was more comfortable overall, and 12/15 (80.0%) stated they would prefer CBBCT for future breast screening.

Across both cohorts, 40/42 participants (95.2%) reported that CBBCT should be more widely available in Australia. Commonly reported barriers included cost, limited geographic access, and lack of Medicare (Australian governments universal health insurance scheme) rebate funding [14].

Participants with previous diagnostic mammography experience more frequently reported moderate-to-extreme mammography pain or discomfort than those with previous routine screening experience (85.2% versus 53.3%, unadjusted *p* = 0.034). This difference did not remain statistically significant after Holm correction for multiple comparisons (adjusted *p* = 0.685). No other subgroup comparisons reached statistical significance before or after adjustment. These findings do not establish equivalence between the groups.

### 3.7. Qualitative Findings

**Figure 1:**
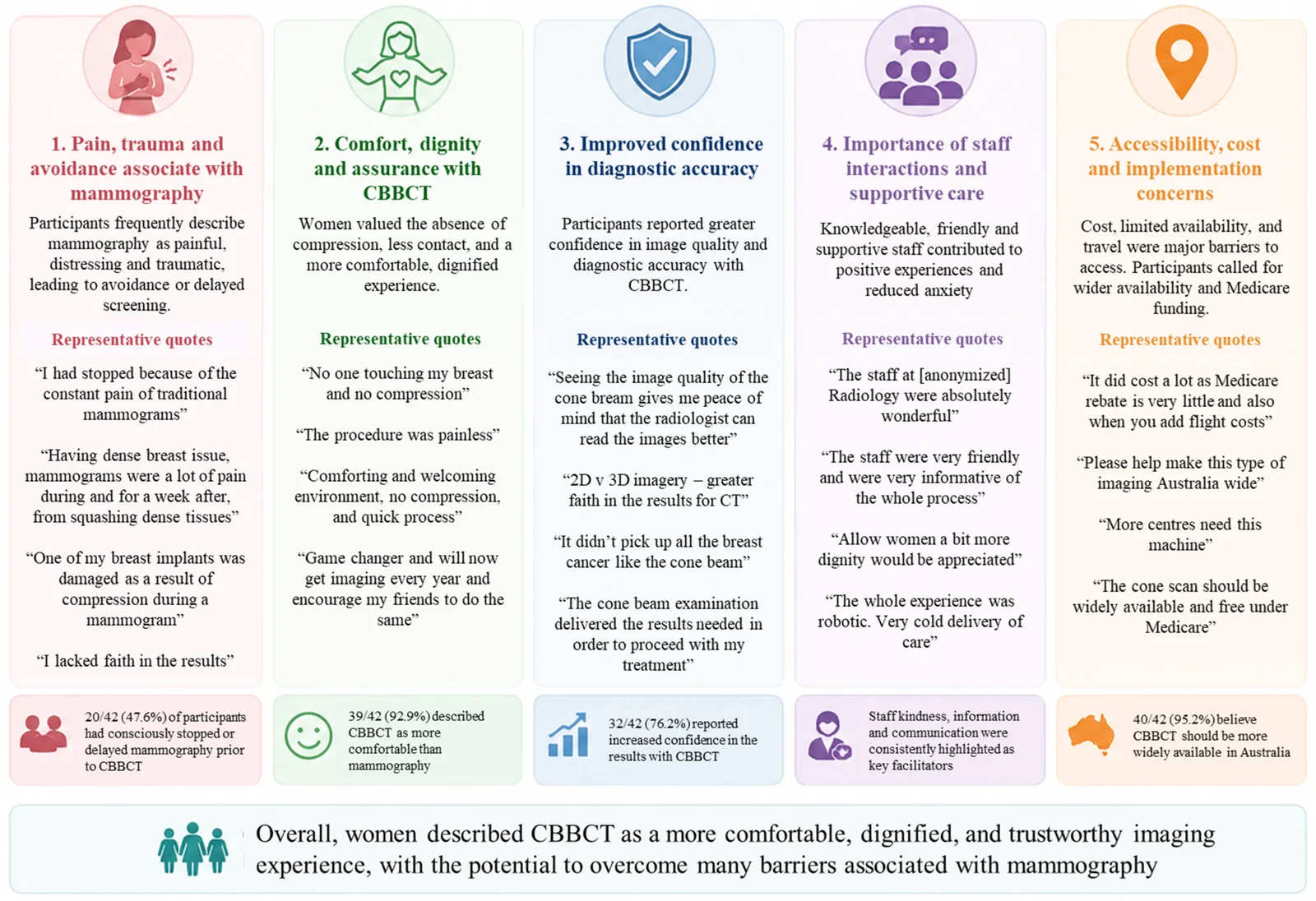
Summary of qualitative themes and representative participant quotations relating to women’s experiences of mammography and cone beam breast CT imaging.

Qualitative analysis identified five major themes: (1) pain, trauma, and avoidance associated with mammography, (2) comfort and dignity associated with CBBCT, (3) improved confidence in diagnostic accuracy, (4) the importance of staff interactions and supportive care, and (5) accessibility and implementation concerns. These themes and representative participant quotations are summarised in Figure 1.

### 3.8. Pain, Trauma, and Avoidance Associated with Mammography

Participants frequently described mammography as painful, distressing, emotionally confronting, and physically uncomfortable. Compression-related discomfort was the dominant concern and was repeatedly linked to delayed or discontinued participation in breast imaging (Table 4).

Several women explicitly linked these experiences to avoidance or delayed participation in future imaging.

### 3.9. Comfort, Dignity, and Reassurance with Cone Beam Breast CT 

Participants overwhelmingly described CBBCT as more comfortable, less invasive, and less distressing than mammography (Table 5).

### 3.10. Improved Confidence in Diagnostic Accuracy

Participants frequently associated CBBCT with improved confidence in image quality, lesion visualisation, and diagnostic reassurance (Table 6).

### 3.11. Staff Interactions and Supportive Care

Positive staff interactions were frequently identified as important contributors to favourable experiences with CBBCT imaging (Table 7).

### 3.12. Accessibility, Cost, and Implementation Concerns

Despite overwhelmingly positive experiences, participants frequently described accessibility and affordability as major barriers to wider adoption of CBBCT imaging (Table 8).

## 4. Discussion

### 4.1. Participant Demographics and Clinical Characteristics

In Australia, mammography is available in two distinct settings: diagnostic and screening. Diagnostic mammography is available for symptomatic patients of all ages and those with a family history of breast cancer and is supported by the Australian government’s universal healthcare funding, Medicare, noting that payment in addition to Medicare (gap payment) may leave patients out of pocket. BreastScreen Australia offers a two-yearly free screening service available to asymptomatic women (only) aged 40 years and over with a target age group of 50–74 years, correlating to the age group most impacted by breast cancer [5]. Women diagnosed in the screening setting with breast cancer thereafter typically attend diagnostic imaging for ongoing follow-up and monitoring [5]. The most represented group, women aged 40–49, are also those not actively recruited by BreastScreen Australia, which may explain their higher representation.

### 4.2. Mammography Experiences

The reported experiences of both cohorts are reflective of what is already known about women’s lived experience of mammography; that women experience fear, pain and discomfort, and place importance on staff interactions and the physical environment [14,15].

Whilst fear of the perceived risk of breast cancer can cause anxiety about having a mammogram [16], pain and discomfort during mammography is reported to be a common but nuanced experience among women [16,17]. Pain is often associated with variability in the compressibility of the breast. Patients with smaller breasts and a low fat-to-glandular tissue ratio, as compared to women with larger breasts and a high fat-to-glandular tissue ratio, have been reported to experience increased pain on compression [18]. Changes in breast composition and compressibility post breast conservation surgery, where scar tissue and breast deformity may impact the procedure, further evidence this concept, where odds were reported as 5.3 times higher for severe pain on compression compared to a non-surgically altered breast [18]. This impact may have influenced the reporting of respondents, where 21.4% identified a previous diagnosis of breast cancer, with 42.9% having surgical intervention. As CBBCT does not utilise compression [19] nor does it involve manual handling of the breasts into position, there is no potential for breast pain from these sources.

### 4.3. Reasons for Discontinuing Mammography

Women reported a high rate of mammography avoidance, with nearly half of those surveyed reporting intentionally stopping or delaying their mammograms. The leading reasons for stopping mammography were pain, discomfort, and fear of compression. Pain during mammography is a well-known precursor to non-engagement in mammographic screening programs [8,20], where women are asymptomatic and may not perceive the threat of cancer as concerning; the decision to avoid the pain of mammography may be an easier choice. Fear of compression, accuracy concerns, and past poor experience all support the need for alternative imaging pathways for these women to ensure they do not completely disengage from imaging and potentially miss early detection of breast cancer. Anticipation of the arrival of CBBCT in Australia is anecdotally known to have influenced the decision of some women not to have mammography.

### 4.4. Reasons for Attending Cone Beam Breast CT Imaging

The pain of compression is one of the primary barriers to participation in mammography. Acknowledging that pain is a unique experience, pain in mammography varies with individual women and radiographic technique. Cone beam breast CT affords a unique imaging experience using no compression. Women in this study chose to attend for CBBCT primarily because it does not use compression as a part of the imaging process. Cone beam breast CT was perceived as a more comfortable and accurate technology in comparison, with women feeling safer and more confident in the results.

### 4.5. Cone Beam Breast CT Imaging Experiences

It is not surprising that women undergoing CBBCT reported high overall satisfaction. as the primary precursor to dissatisfaction, pain, was eliminated from the procedure. Indeed, minimal to no pain was reported outside of the minor physical challenge for these women of accessing the CBBCT table, shoulder discomfort, and mobility issues. This is an important observation as the physicality of patients will indeed limit their ability to access CBBCT. Currently, mammography of women in wheelchairs remains, for the woman, physically and for the radiographer technically challenging but possible for most women [21]. There is no such capability with CBBCT outside of utilising lifting devices and having staff available to roll patients into position. The authors are not aware of this approach currently being implemented in practice.

### 4.6. Comparison Between Mammography and Cone Beam Breast CT

The study has identified that CBBCT is overwhelmingly preferred to mammography in this cohort of women. This preference may translate to others and is an important consideration for screening programs where poor participation continues to compromise the efficacy of the Australian program.

Women in this study strongly advocated for the widespread availability of CBBCT in Australia, and it is the view of the authors that whilst it is acknowledged that CBBCT can not service all women, the majority who can benefit from a more positive and superior imaging experience should. As validation of the technology is progressed in the Australian setting, it is hoped a more generalised adoption of the technology will occur, and the current barriers of cost, availability and Medicare rebate will be overcome.

### 4.7. Qualitative Findings

The open-ended questions in this study support the qualitative data with themes reinforcing the pain and avoidance of mammography, the comfort and improved dignity (mostly) of CBBCT, increased diagnostic confidence and implementation barriers. Women also noted the value of supportive staff in the imaging process, which is at the core of person-centred mammographic imaging.

### 4.8. Pain, Trauma, and Avoidance Associated with Mammography

Mammography is universally considered to be associated with discomfort and pain, pain being the most studied outcome of mammography in the literature [22]. Participants, through their open-ended responses again described mammography as painful, distressing, emotionally confronting, and physically uncomfortable, which is unsurprising.

Some trauma was related to breast implant damage during mammography. The risk of damage or rupture of breast implants during CBBCT is negated due to the absence of compression. Current best practice for imaging women with breast implants using mammography includes a non-compressed imaging series (four views) and an implant-displace (ID/Eklund technique) (four views) series using compression [23,24], although there is known variability across the nation [25] and ongoing debate as to whether this number of views (eight) can be reduced. Even when the ID technique is correctly undertaken, there is still risk to the implant, particularly where the implant is old and its integrity compromised [26,27]. Cone beam breast CT mean glandular dose has been reported to be within the range of that from conventional 2D mammography [28]. The additional ID views required for implant imaging in 2D mammography add incremental dose to the patient, something that does not impact CBBCT, as the imaging protocol requires each breast to only be imaged once to complete a routine examination. Because CBBCT allows visualisation of the breast from the nipple to the chest wall [28], more breast tissue is visible to the radiologist in all women, including those with breast implants, improving the potential for breast cancer detection. The isotropic images of CBBCT also increase diagnostic accuracy due to the ability to accurately reconstruct images in any plane without loss of resolution and with no tissue overlap [28].

### 4.9. Comfort, Dignity, and Reassurance with Cone Beam Breast CT

The defining factor in the preference for CBBCT was the absence of breast compression. The removal of physical discomfort combined with an overall less invasive procedure supported reduced patient anxiety and resulted in CBBCT being identified as the preferred imaging method to traditional mammography. Lack of dignity due to the need to undress and have the radiographer manually position the breasts for mammography has also been removed, resulting in an increased willingness to engage in future breast imaging.

### 4.10. Improved Confidence in Diagnostic Accuracy

With the ease of access to the internet and large language models, the authors hypothesize that women may have conducted their own investigations as to the efficacy of CBBCT before seeking referral. This may have impacted the breadth of opinion across image superiority, better image quality, and diagnostic capability that was further linked to lack of pain. Pain was negatively associated with “unclear results,” which may be an outcome of a previously difficult mammogram. The belief in improved diagnostic accuracy may be resultant from discussions with their referrer and the referrer’s understanding of the benefits of the technology.

### 4.11. Staff Interactions and Supportive Care

Breast imaging is highly emotive, and the importance of good communication and empathy across the imaging pathway is critical to support imaging and the patient experience. It is interesting that there was reporting of “*robotic and cold delivery of care*” and a “*lack of patient dignity*”. When one considers the substantive change in the process of imaging being the negation of one-on-one hands-on positioning, breast manipulation, and body contact between the radiographer and client in mammography to self positioning/manipulation, (getting on one’s hands and knees and lowering oneself onto the CBBCT table), with verbally directed positioning (as opposed to direct touching by the radiographer), this study may have revealed a new and potentially negative aspect of the patient experience not previously considered.

### 4.12. Accessibility, Cost, and Implementation Concerns

Unlike mammography and other adjunct breast imaging including digital breast tomosynthesis (DBT), contrast enhanced mammography (CEM), ultrasound (US), magnetic resonance imaging (MRI) and breast biopsy which attract a Medicare rebate, the recency of CBBCT means that the Australian Government is yet to fully assess the efficacy of the technology to add this imaging to the Medicare Benefits schedule. The technology is also yet to be validated in Australia for use in the free breast screening programme. This means that women are currently left to pay the full cost of CBBCT imaging and gap payments for adjunct imaging such as ultrasound.

There is clear support for the technology, and those women who can afford imaging have voted with their feet and travelled vast distances to attend. Accessibility will inevitably increase along with increased installation, government rebates, and adoption by the medical community as an alternative to traditional mammography.

Ergonomic issues raised around ease of getting onto the CBBCT table and its overall comfort may require further consideration by the vendor. However, comments may be a reflection of individual participant body habitus and ability rather than a common experience for all. The direct cause is outside the scope of this study.

### 4.13. Limitations

The study has limitations, including having a small response rate to the survey, being only 9% of those scanned during the study period. At the time of the research, there was only one CBBCT unit in Australia, creating a risk of selection bias. Patients may represent a cohort with preexisting dissatisfaction towards conventional mammography and high positive expectations for CBBCT. The results therefore also reflect only a small, single-site sample; results may not be generalisable. Non-response bias may further limit interpretation, as the experiences of respondents may differ from those of women who did not participate. Participants retrospectively recalled their mammography experiences, introducing potential recall bias. Differences in examination timing, clinical circumstances, and staff interactions may also have influenced comparisons with CBBCT. The subgroup analyses were exploratory, and the small, unequal groups limited statistical power to detect differences. No comparisons remained statistically significant after Holm correction for multiple testing. These findings do not establish equivalence between the diagnostic and screening groups. The survey measured perceived diagnostic confidence rather than diagnostic accuracy. Preferences for future CBBCT imaging also do not demonstrate subsequent attendance or improved screening adherence. The study remains important as it provides a first look at Australian women’s experience with CBBCT.

## 5. Conclusions

These preliminary findings highlight the substantial pain, discomfort, and anxiety associated with mammography, which contributed to delayed or discontinued imaging among many participants. In contrast, CBBCT was perceived as a comfortable, well-tolerated, and compression-free alternative, with most women preferring it over mammography. Participants strongly supported wider access to CBBCT despite current barriers related to cost and availability.

## Figures and Tables

**Table 1:** Participant demographic and clinical characteristics.

Characteristic	Total n (%)	Diagnostic Setting n (%)	Routine Screening n (%)
Total participants	42 (100)	27 (64.3)	15 (35.7)
Previous diagnosis of breast cancer	9 (21.4)	5 (18.5)	4 (26.7)
Previous breast surgery	18 (42.9)	11 (40.7)	7 (46.7)
Breast implants	12 (28.6)	7 (25.9)	5 (33.3)
Family history of breast cancer	22 (52.4)	14 (51.9)	8 (53.3)
Consciously stopped mammography prior to CBBCT	20 (47.6)	14 (51.9)	6 (40.0)
Age 40–49 years	16 (38.1)	9 (33.3)	7 (46.7)
Age 50–59 years	12 (28.6)	9 (33.3)	3 (20.0)
Age 60–69 years	9 (21.4)	6 (22.2)	3 (20.0)
Age ≥70 years	4 (9.5)	3 (11.1)	1 (6.7)
Age <30 years	1 (2.4)	1 (3.7)	0 (0.0)

**Table 4:** Pain, trauma, and avoidance associated with mammography.

Subject	Comment
Several women described longstanding fear and anxiety surrounding mammography appointments.	“*I always hated the time of year my compression mammogram was due because of the pain involved*”.
	“*I had stopped because of the constant pain of traditional mammograms*”.
Pain associated with dense breast tissue was commonly reported.	“*Having dense breast tissue, mammograms were a lot of pain during and for a week after from squashing dense tissue*”.
Several participants described concerns that mammography did not adequately visualise dense breasts or all breast tissue.	“*The new method no compression and captured all my breast tissue as felt like mammogram missed some area near the armpit*”.
Participants also described experiences of emotional distress and loss of confidence in mammography systems	“*Dense breasts such as mine don’t show clear results on mammogram*”.
	“*I lacked faith in the results*”.
Another participant described frustration following repeated investigations:	“*Getting a ‘everything was ok see you in 2 years’ letter from BSQ (sic BreastScreen Queensland) following mammogram when I had lots of large lumps in my breasts*”.
Some participants described traumatic experiences associated with breast implants and previous surgery.	“*One of my breast implants was damaged as a result of compression during a mammogram examination*”.
	“*Each mammogram I had done after that became more and more painful*”.
Another participant with a history of breast cancer described mammography after surgery as	“*Very painful and upsetting*”.

**Table 5:** Comfort, dignity, and reassurance with cone beam breast CT.

Subject	Comment
The absence of breast compression was repeatedly identified as the most important contributor to improved experiences.	*“No one touching my breast and no compression”.*
	*“Squeezing or compression of breasts doesn’t occur with CT”.*
Women frequently described the examination as painless and significantly less anxiety provoking.	*“The procedure was painless”.*
	*“The ease of scan and the clear results have significantly decreased my anxiety regarding cancer”.*
Participants also associated CBBCT with greater dignity and less confronting positioning.	*“Felt I was treated with more dignity”.*
	*“Comforting and welcoming environment, no compression and quick process without poking, prodding, squashing and painful compression”.*
For many participants, the experience fundamentally changed their willingness to engage in future breast imaging.	*“Game changer and will now get imaging every year and encourage my friends to do the same”.*
	*“Mammograms were painful and didn’t show clear results like ultrasound and CT imaging do”.*
	*“Love it, will always do it again”.*
Several participants described the technology as transformative	*“This new technology is fabulous!”*
	*“Such a great improvement to this important screening”.*

**Table 6:** Improved confidence in diagnostic accuracy.

Subject	Comment
Women with dense breast tissue particularly valued the perceived three-dimensional imaging capabilities of CBBCT.	“*Seeing the image quality of the cone beam gives me peace of mind that the radiologist can read the images better*”.
	“*2D v 3D imagery—greater faith in the results for CT*”.
Several women described concerns that mammography had previously failed to identify abnormalities or had required repeated recalls.	“*It didn’t pick up all the breast cancer like the cone beam*”.
Participants also described reassurance associated with clearer imaging and more confidence in outcomes.	“*The cone beam examination was painless and delivered the results needed in order to proceed with my treatment*”.

**Table 7:** Staff interactions and supportive care.

Subject	Comment
Participants commonly described staff as supportive, calming, knowledgeable, and informative.	“*The staff …were absolutely wonderful*”.
	“*The staff were very friendly and were very informative of the whole process*”.
Several women specifically highlighted reassurance and communication as important factors contributing to comfort during imaging.	“*Friendly staff my Dr said the radiologist was excellent*”.
A small number of women reported concerns relating to dignity, empathy, and communication.	“*The whole experience was robotic. Very cold delivery of care*”.
	“*Allow women a bit more dignity would be appreciated*”.

**Table 8:** Accessibility, cost, and implementation concerns.

Subject	Comment
Several women reported substantial out-of-pocket costs, limited-service availability, and long-distance travel requirements.	“*It did cost a lot as Medicare rebate is very little (sic for CBBCT and adjunct imaging) and also when you add flight costs as well*”.
	“*All up it cost me $AU700 out of pocket*”.
Participants strongly advocated for broader implementation of the technology throughout Australia.	“*Please help make this type of imaging Australia wide*”.
	“*More centres need this machine*”.
Calls for improved Medicare funding and equitable access were also common	“*The cone scan should be widely available and free under Medicare*”.
	“*The gov to fund the purchase and implementation of these machines for all women who require them*”.
Participants additionally highlighted practical considerations regarding physical accessibility and positioning.	“*Maybe a sturdy rail to hold when climbing*”.
	“*The cone scan table needs to be softer for positioning instead of lying on hard plastic*”.

## Data Availability

The raw data supporting the conclusions of this article will be made available by the authors on request.

## Supplementary Materials

The following supporting information can be downloaded at: https://www.mdpi.com/article/10.3390/jcm15208085/s1, Table S1: Raw Dataset.

## Abbreviations

The following abbreviations are used in this manuscript:

CBBCT	Cone Beam Breast Computed Tomography
CEM	Contrast Enhanced Mammography
DBT	Digital Breast Tomosynthesis
ID	Implant-Displaced
MRI	Magnetic Resonance Imaging
US	Ultrasound
